# Reliability and validity of the ICF core elements scale for swallowing function assessment in stroke patients: a single-center study from Hunan, China

**DOI:** 10.3389/fneur.2026.1863539

**Published:** 2026-10-07

**Authors:** Yi Dong, Hao Tan, Junrong Chen, Pei Qin, Yuqi Yin, Xi Tao

**Affiliations:** 1 Department of Rehabilitation Medicine, Hunan Provincial People's Hospital, Hunan Normal University, Changsha, Hunan, China; 2 Department of Integrated Traditional Chinese and Western Medicine, Hunan Provincial People's Hospital, Hunan Normal University, Changsha, Hunan, China; 3 Department of Nursing, Changde Vocational Technical College, Changde, Hunan, China; 4 Vascular Depression Diagnosis, Treatment, and Rehabilitation Technology Innovation Center of Changsha, Changsha, Hunan, China

**Keywords:** dysphagia, International Classification of Functioning, Disability and Health, stroke, reliability, validity

## Abstract

**Objective:**

To evaluate the reliability and validity of the 21-item ICF Core Category Scale for Dysphagia in Stroke (ICCSDS) in assessing swallowing disorders in Chinese stroke patients.

**Methods:**

Six experienced rehabilitation physicians evaluated the content validity of the scale. Two raters (a rehabilitation physician and a swallowing therapist) received standardized training. Rehabilitation physicians screened and enrolled 80 patients with suspected swallowing difficulties using the Toronto Bedside Swallowing Screening Test. A swallowing therapist subsequently performed the initial ICCSDS assessment using the Gugging Swallowing Screen (GUSS) as a practical reference standard. Four hours later, a rehabilitation physician conducted a second ICCSDS assessment at the same site to evaluate interrater reliability.

**Results:**

Significant differences were observed between the high-score and low-score groups for all the ICCSDS items (all *p* < 0.01). The Cronbach’s α coefficients for assessments performed by the rehabilitation physician and swallowing therapist were 0.864 and 0.829, respectively. The S-CVI value was 0.978, and the I-CVI values ranged from 0.83 to 1.00. Exploratory factor analysis revealed that the Kaiser–Meyer–Olkin (KMO) values for assessments by the rehabilitation physician and the swallowing therapist were 0.887 and 0.848, respectively (both *p* < 0.01), with cumulative variance contributions of 75.69 and 73.65%, respectively. When the GUSS was used as the practical reference standard, the criterion-related validity coefficients of the ICCSDS assessed by the rehabilitation physician and the swallowing therapist were 0.790 and 0.683, respectively (both *p* < 0.01). Interrater reliability analysis revealed an ICC (2, 1) of 0.924 and an ICC (2, k) of 0.961. The weighted kappa coefficients exceeded 0.80 for 14 items, reached 0.799 for one item, and ranged from 0.420 to 0.547 for six items.

**Conclusion:**

The 21-item ICCSDS demonstrated acceptable internal consistency, content validity, and interrater reliability in Chinese patients with poststroke dysphagia. The preliminary evidence from the criterion-related validity analysis supports its association with clinical bedside assessment tools.

## 1 Introduction

Stroke has a high incidence in China and is associated with high rates of disability and mortality. Recent epidemiological reports indicate that the incidence of poststroke dysphagia ranges from 22 to 65%, with figures varying across different studies (1–3). As a common poststroke functional impairment, dysphagia not only leads to malnutrition and aspiration pneumonia but also significantly increases anxiety and depression, reduces quality of life, and consequently increases the risk of mortality (4, 5). Therefore, a timely and comprehensive assessment of swallowing function is crucial for reducing complications, guiding swallowing therapy, and promoting comprehensive rehabilitation (3). Currently, commonly used clinical assessment methods include instrumental and scale-based evaluations (6). Instrumental assessments, such as fiberoptic endoscopic evaluation of swallowing (FEES) and videofluoroscopic swallowing studies, offer precision and objectivity. However, these techniques require specialized equipment and facilities, involve high costs, and are difficult to perform repeatedly. In contrast, scale-based assessment is currently the mainstream approach, which can be implemented in primary care hospitals and offers advantages such as bedside operation, simplicity, ease of use, and low cost.

Existing assessment scales focus primarily on specific aspects of dysphagia severity, treatment efficacy, or quality of life. Tests such as the water swallowing test, repetitive saliva swallowing test, and Toronto Bedside Swallowing Screening Test (TOR-BSST) allow rapid preliminary screening and are simple to administer (7). However, these methods have relatively low sensitivity; they cannot detect the risk of silent aspiration, nor can they localize the specific site of the swallowing impairment or assess the impact of different food consistencies. Although scales such as the Standardized Swallowing Assessment, Functional Oral Intake Scale, Gugging Swallowing Screen (GUSS), and volume-viscosity swallow test assess the influence of different food consistencies on swallowing function, they do not focus on the dimension of patient quality of life (8, 9). Scales such as the Eating Assessment Tool and the Swallowing Quality of Life Questionnaire address the quality-of-life dimension (10, 11), but their guidance for specific types of swallowing therapy remains insufficient. Furthermore, such scales are relatively subjective, making them difficult to administer to patients with cognitive or communication impairments (e.g., aphasia, attention-deficit disorder), and they are less reliable. These scales generally evaluate only one aspect of swallowing dysfunction and lack a comprehensive assessment of social activities, participation capacity, and particularly environmental factors, which may lead to insufficient risk assessment (12).

In 2001, the World Health Organization officially promulgated the “International Classification of Functioning, Disability, and Health” (ICF). This classification is widely recognized for its ability to comprehensively assess a patient’s functional status at three levels: body, individual, and societal. It has become an internationally accepted language and conceptual framework. Although the ICF contains numerous components, its core categories—including body functions and structures, activities and participation, and environmental factors—are sufficient to provide specific directions and goals for patient functional rehabilitation, thereby improving the quality of healthcare services within a limited clinical time (13). In 2007, Professor Threats extracted 60 items from the comprehensive ICF categories specifically for swallowing dysfunction to facilitate global promotion. However, these 60 items are overly broad, resulting in lengthy clinical assessments. Additionally, the definitions of some items are too abstract, making them difficult for both assessors and patients to understand and hindering their adoption in the fast-paced routine of daily clinical work.

In 2015, considering the characteristics of swallowing disorders and the cultural context of the Chinese population, we distilled 21 core ICF categories for poststroke dysphagia from the list provided by Professor Threats (14). In recent years, we have developed these categories into the ICF Core Category Scale for Dysphagia in Stroke (ICCSDS), which is used to assess patients’ swallowing function across multiple levels, including body structure and function, activities and participation, and environmental factors. We subsequently analyzed the reliability and validity of this scale, providing a basis for promoting and applying it in the Hunan region of China.

## 2 Materials and methods

### 2.1 Study subjects

This study included 80 stroke patients admitted to the Department of Rehabilitation Medicine or the Department of Neurology at Hunan Provincial People’s Hospital (The First Affiliated Hospital of Hunan Normal University). This study was approved by the Ethics Committee of Hunan Provincial People’s Hospital (The First Affiliated Hospital of Hunan Normal University) (Approval No.: 2024-21). Written informed consent was obtained from all patients or their family members prior to assessment.

The inclusion criteria were as follows: (1) met the diagnostic criteria for various cerebrovascular diseases, with brain infarction or cerebral hemorrhage confirmed by head CT or MRI; (2) were conscious; and (3) were able to complete the TOR-BSST and were diagnosed with swallowing dysfunction.

The exclusion criteria were as follows: (1) the presence of dysphagia prior to stroke onset; (2) the presence of psychogenic dysphagia caused by stroke; (3) poststroke dementia; (4) severe cardiopulmonary insufficiency; (5) the presence of pulmonary infection at the time of assessment; and (6) having undergone tracheostomy.

### 2.2 Research instruments

#### 2.2.1 TOR-BSST scale

This scale is an effective bedside tool for rapid screening of swallowing function. The scale is specifically divided into four steps. The first step is a prewater assessment, which involves observing the patient’s voice quality and head or tongue movement. The second step is the water-swallowing test, which involves observing the presence of three major symptoms: choking/coughing, voice change, and drooling. The third step is a postwater voice assessment that determines whether the voice is abnormal 1 min after the test concludes. The fourth step involves evaluating the results of the task assessment. Research has shown that this scale typically achieves a sensitivity of more than 85% and a specificity of approximately 80% for screening dysphagia in stroke patients, both of which are superior to those of traditional unstructured clinical assessments (7). Furthermore, the pathological mechanisms underlying dysphagia are diverse and complex, making it difficult to fully elucidate its pathological features solely through clinical observation and simple tests. This difficulty also limits the depth and precision of TOR-BSST assessment (15).

#### 2.2.2 ICCSDS

This core category set comprises 21 items (14), including 13 for body functions, 1 for body structures, 3 for activities and participation, and 4 for environmental factors. Items related to activities and participation are further subdivided into P (performance: completing the task with suitable assistive devices or caregiver assistance) and C (capacity: completing the task without assistive devices or caregiver assistance) (Supplementary Table 1). Patients are assessed through questionnaires and clinical examinations.

*ICCSDS scoring criteria*: The ICF categories were rated using a five-point Likert scale, with the following anchor definitions: 0 = no problem (no swallowing-related problems, 0–4% impairment); 1 = mild problem (5–24%); 2 = moderate problem (25–49%); 3 = severe problem (50–95%); and 4 = complete problem (96–100%). Higher scores indicate more severe swallowing-related functional impairment. In the present study, for the convenience of statistical analysis, environmental factor items (e110, e340, e410, and e580) were coded such that positive values (1–4) represented barriers, whereas negative values (−1 to −4) represented facilitators. Negative values were entered directly as negative scores and included in the total score together with positively scored items. Codes 8 (not specified) and 9 (not applicable) were treated as missing values in the statistical analyses and were excluded from the calculation of both the corresponding item scores and the total score.

*Total score calculation*: The theoretical total score of the ICCSDS was calculated as the sum of the scores across all scoring units. There were 17 positively scored units in total (13 b-category units + 1 s-category unit + 3 d-category Performance [P] subitems; the 3 d-category Capacity [C] subitems were not included in the total score), yielding a theoretical score range of 0–68. The four environmental factor items had scores ranging from −16 to +16. Scores from these components could be combined to derive an overall score, while component-specific scores could also be reported separately to reflect the distribution of impairments across different functional dimensions.

*Assessment procedure*: Patients were independently assessed using questionnaire-based evaluation and clinical examination.

#### 2.2.3 GUSS scale

This bedside assessment tool evaluates swallowing function and is widely used in clinical practice because of its simplicity, safety, and effectiveness (16). The GUSS assessment process comprises two parts (17). (1) Indirect Swallowing Test (5 points): The patient sits, actively coughs or clears the throat, can swallow saliva, and shows no drooling, hoarseness, or other voice changes. If the above process is completed successfully, the assessment proceeds to the second part; otherwise, it is stopped. (2) Direct Swallowing Test: Testing is conducted by varying food consistency sequentially using pureed, liquid, and solid foods. Each type of food consistency is worth 5 points. Observations include swallowing actions, coughing, drooling, voice changes, etc., and are scored accordingly. The total GUSS score is 20 points. A score of 9 points or less indicates swallowing dysfunction. This assessment recommends the initiation of semisolid foods and then transitioning to liquids and solids. This approach not only reduces the risk of aspiration and makes the assessment process safer but also comprehensively assesses the patient’s ability to swallow food of varying consistencies. It is particularly sensitive to silent aspiration (aspiration without a significant cough response) and demonstrates high reliability and validity (18). Notably, the GUSS is a bedside screening tool rather than an instrumental diagnostic gold standard, such as videofluoroscopic swallowing study (VFSS) or FEES. However, given its convenience and high safety, it was used in the present study as a pragmatic comparator for evaluating criterion-related validity.

### 2.3 Research methods

*Step 1*: Six rehabilitation experts with more than ten years of clinical experience in swallowing assessment were selected to score the ICCSDS content. Specifically, the experts were required to score each category on the basis of the relevance of the assessed content to stroke-related dysphagia. The scoring used a 4-point scale: 1 point indicated not relevant, 2 points indicated weakly relevant, 3 points indicated strongly relevant, and 4 points indicated highly relevant. Consultation with these authoritative experts confirmed that the assessment categories and content of this scale are reasonable, comprehensive, and representative.

*Step 2*: Professional training was provided to the assessors. The assessors in this study were a deputy chief physician from the Department of Rehabilitation Medicine with 15 years of work experience and a swallowing therapist with 10 years of work experience. Before the assessment tests were conducted, both individuals received 6 h of systematic training on the quantitative standard scoring of the ICCSDS. The training focused on the standardization of operational definitions, anchor points for severity levels, and scoring criteria for each item to reduce discrepancies in item interpretation among the raters.

*Step 3*: Standardized assessment was conducted. First, rehabilitation physicians used the TOR-BSST to screen patients with suspected dysphagia for enrollment. Second, a swallowing therapist performed the GUSS assessment as an external reference standard for criterion-related validity testing, followed by the first ICCSDS assessment. Four hours later, a rehabilitation physician conducted the second ICCSDS assessment at the same location. The two assessors were not allowed to communicate the assessment results between the two evaluations. Notably, the two ICCSDS assessments were performed by different assessors; therefore, this procedure was designed primarily to evaluate interrater reliability. The research flowchart is shown in Figure 1.

**Figure 1:**
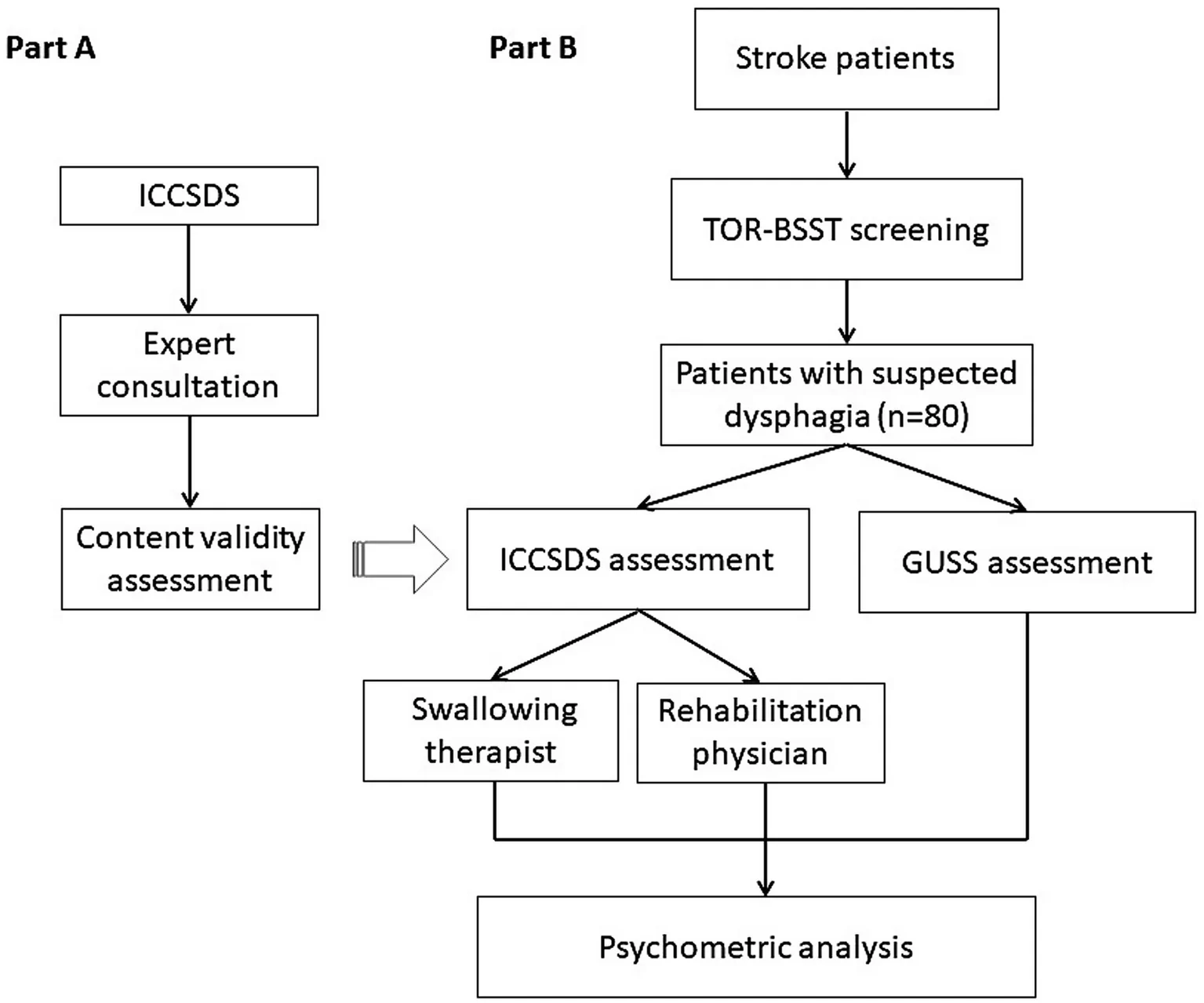
Study flow chart. This study consisted of two parts. Part **A**: Scale development/content validity. Part **B**: Clinical psychometric validation. GUSS, Gugging Swallowing Screen; ICCSDS, ICF Core Category Scale for Dysphagia in Stroke; TOR-BSST, Toronto Bedside Swallowing Screening Test.

### 2.4 Statistical methods

All the data were entered into Excel, and SPSS 26.0 was used for descriptive statistics, item analysis, and reliability and validity testing. Among these, item analysis included the critical ratio method and degree-of-dispersion analysis. The critical ratio method involved ranking the total scores and then selecting the highest 27% and lowest 27% of the total scores to form high-score and low-score groups, respectively. An independent samples *t* test was then performed on the total scores of the two groups. The presence of significant differences between the high- and low-score groups across all items indicates that the items have good discriminative power (19).

Reliability and validity testing included the following: (1) Content validity was assessed using the item-level content validity index (I-CVI) and the scale-level content validity index (S-CVI). Generally, an I-CVI ≥ 0.78 and an S-CVI ≥ 0.9 are considered indicators of good content validity (20, 21). (2) Reliability analysis was conducted using Cronbach’s alpha coefficient. A Cronbach’s alpha coefficient between 0.60 and 0.80 suggests good internal consistency (22). (3) Construct validity was determined using exploratory factor analysis (EFA). (4) Criterion-related validity was assessed using Pearson product–moment correlation analysis. (5) Interrater reliability was evaluated using the intraclass correlation coefficient (ICC) to assess the agreement between the scores assigned by the two raters, with the corresponding 95% confidence intervals reported. In addition, item-level weighted kappa coefficients were calculated to evaluate the degree of agreement for each item, providing a comprehensive assessment of interrater consistency.

## 3 Results

### 3.1 General information

A total of 80 stroke patients, including 62 males (77.50%) and 18 females (22.50%), were screened. Among them, 49 patients (61.30%) were over 60 years old, and 31 (38.70%) were 60 years or younger. The average patient age was 63.96 ± 12.10 years, and the average disease duration was 7.27 ± 16.26 months. Specifically, 5 patients (6.25%) had a disease duration of less than 6 months, while 75 patients (93.75%) had a disease duration of 6 months or longer. The average years of education was 10.18 ± 4.74 years. Among these individuals, 55 (68.75%) had more than 6 years of education, 19 (23.75%) had 0 to 6 years of education (inclusive), and 6 (7.50%) had no formal education (0 years).

Additionally, the proportions of patients with cerebral hemorrhage (*n* = 29), cerebral infarction (*n* = 46), and subarachnoid hemorrhage (*n* = 5) were 36.25, 57.50, and 6.25%, respectively. Among the stroke patients, 63 experienced the disease once, 16 experienced it twice, and only 1 experienced it three times (Table 1).

**Table 1:** General information of stroke patients with dysphagia.

Variables		Frequency (*n*)	Constituent ratio (%)
Sex	Male	62	77.50
	Female	18	22.50
Age (years)	≤ 60	31	38.75
	> 60	49	61.25
Education (years)	> 6	55	68.75
	1–6	19	23.75
	0	6	7.50
Diagnostic classification	Cerebral hemorrhage	29	36.25
	Cerebral infarction	46	57.50
	Subarachnoid hemorrhage	5	6.25
Course of disease (months)	< 6	5	6.25
	≥ 6	75	93.75
Number of strokes	1	63	79.79
	2	16	20.00
	3	1	1.25

### 3.2 Item dispersion of the ICCSDS

From an overall perspective of dispersion, the standard deviation (SD) of the scores for each ICCSDS item assessed by the rehabilitation physicians ranged from 0.93 to 1.84, while that assessed by the swallowing therapists ranged from 0.79 to 1.71. These findings that the sensitivity of all individual items is good. The SDs for all categories were significantly greater than 0.5, indicating sufficient dispersion. These findings imply that the ICCSDS has good sensitivity and item discrimination ability; thus, it can effectively distinguish individual differences among patients.

At the item level, the two raters demonstrated a high degree of consistency in the distribution of scores for most items, although discrepancies were observed for some items. With respect to the items with the greatest score variability, the rehabilitation physician reported the highest SD of 1.84 for category b1302 (Appetite), whereas the swallowing therapist reported the highest SD of 1.71 for category e110 (Products or substances for personal consumption). With respect to the items with the lowest score variability, the rehabilitation physician reported the lowest SD of 0.93 for category e580 (Health services, systems, and policies), whereas the swallowing therapist reported the lowest SD of 0.79 for category s320 (Structure of the mouth). Notably, the scores for categories e110, e340, e410, and e580 (all environmental factors) were consistently negative. These findings indicate that both raters generally and consistently considered these environmental factors to function as “facilitators” (Table 2).

**Table 2:** Scores of the 21 Core ICCSDS Items by rehabilitation physicians and swallowing therapists.

ICF code	Rehabilitation physician	Swallowing therapist
b117	2.26 ± 1.62	1.81 ± 1.62
b1301	2.54 ± 1.50	1.94 ± 1.58
b1302	2.40 ± 1.84	1.95 ± 1.49
b1303	2.55 ± 1.47	1.74 ± 1.53
b140	2.45 ± 1.45	2.41 ± 1.38
b156	2.32 ± 1.48	2.31 ± 1.43
b1644	2.42 ± 1.45	2.40 ± 1.40
b2102	2.36 ± 1.52	2.30 ± 1.55
b250	2.33 ± 1.59	2.34 ± 1.54
b510	2.86 ± 1.39	2.84 ± 1.44
b5101	2.75 ± 1.30	2.55 ± 1.35
b5102	2.75 ± 1.26	2.35 ± 1.32
b51050	2.71 ± 1.32	2.71 ± 1.34
s320	1.19 ± 1.21	0.61 ± 0.79
d550P	1.18 ± 1.12	1.20 ± 1.10
d550C	2.18 ± 1.40	2.70 ± 1.39
d560P	1.00 ± 1.39	1.06 ± 1.35
d560C	2.90 ± 1.46	2.63 ± 1.39
d630P	2.81 ± 1.37	2.84 ± 1.27
d630C	3.21 ± 1.40	3.24 ± 1.30
e110	−2.45 ± 1.71	−2.48 ± 1.71
e340	−3.49 ± 1.06	−3.44 ± 1.03
e410	−2.82 ± 1.09	−3.00 ± 0.98
e580	−3.19 ± 0.93	−3.27 ± 0.83

All the data are presented as the mean ± SD.

ICCSDS, ICF Core Category Scale for Dysphagia in Stroke; P, performance; C, capacity.

### 3.3 Critical ratio analysis

The total scores of the ICCSDS items were ranked, and the top 27% of the total scores were compared with the bottom 27% using an independent samples t test. The results revealed statistically significant differences for all the items between the high-scoring and low-scoring groups, as assessed by both the rehabilitation physician and the swallowing therapist (all *p* < 0.01), indicating good item discrimination.

### 3.4 Reliability testing

The internal consistency analysis revealed that the Cronbach’s α coefficients for the ICCSDS assessments performed by the rehabilitation physician and swallowing therapist were 0.864 and 0.829, respectively, indicating good to excellent internal consistency of the ICCSDS when it was used by different assessors. The two α coefficients were highly similar, further suggesting the good homogeneity and stability of the scale items across assessors with different professional backgrounds (Table 3).

**Table 3:** Reliability and validity testing of the ICCSDS.

Indicators		Rehabilitation physician (95% CI)	Swallowing therapist (95% CI)
ICCSDS score		35.04 ± 17.51	35.86 ± 17.79
Cronbach’s α		0.864 (0.840 ~ 0.882)	0.829 (0.749 ~ 0.855)
Interrater reliability	ICC (2, 1)	0.924 (0.880 ~ 0.952)	
	ICC (2, k)	0.961 (0.936 ~ 0.975)	
Construct Validity**	KMO	0.887	0.848
	Bartlett’s test for sphericity	1621.280	1179.518
Criterion-related validity**		0.790 (0.689 ~ 0.861)	0.683 (0.543 ~ 0.786)

**p < 0.01.

ICCSDS, ICF Core Category Scale for Dysphagia in Stroke; KMO, Kaiser–Meyer–Olkin; ICC, intraclass correlation coefficient.

Interrater reliability analysis revealed a high level of agreement between the two raters, with an ICC (2, 1) of 0.924 and an ICC (2, k) of 0.961, indicating strong consistency in the ICCSDS total scores between the two raters (Table 3).

The results of the interrater agreement analysis revealed that the weighted kappa coefficients (exceeding 0.80 for 14 items) for the ICCSDS items were generally high, indicating very strong interrater agreement; one item showed substantial agreement (0.799), while six items showed moderate agreement (0.420–0.547), suggesting that the scoring consistency for these items requires further improvement (Table 4).

**Table 4:** Weighted kappa coefficients for individual ICCSDS items.

ICF code	Weighted Kappa	95% CI
b117	0.509	0.336 ~ 0.682
b1301	0.799	0.719 ~ 0.878
b1302	0.829	0.729 ~ 0.930
b1303	0.834	0.751 ~ 0.917
b140	0.959	0.938 ~ 0.980
b156	0.937	0.909 ~ 0.965
b1644	0.969	0.948 ~ 0.989
b2102	0.976	0.960 ~ 0.992
b250	0.982	0.969 ~ 0.995
b510	0.437	0.227 ~ 0.648
b5101	0.421	0.241 ~ 0.601
b5102	0.420	0.254 ~ 0.587
b51050	0.979	0.962 ~ 0.995
s320	0.800	0.681 ~ 0.920
d550P	0.938	0.901 ~ 0.976
d560P	0.976	0.958 ~ 0.995
d630P	0.964	0.943 ~ 0.984
e110	0.987	0.975 ~ 0.999
e340	0.965	0.934 ~ 0.996
e410	0.547	0.305 ~ 0.788
e580	0.454	0.207 ~ 0.702

Because the C-related items were not included in the calculation of the total score, weighted kappa coefficients were not calculated for these items.

ICCSDS, ICF Core Category Scale for Dysphagia in Stroke; P, performance; C, capacity.

### 3.5 Validity testing

#### 3.5.1 Content validity

The content validity of the ICCSDS was evaluated using an expert consultation scoring method. Six experts were invited to rate the relevance of the content. The I-CVI was 1 for 18 items. For three items—b1303 (Craving), b156 (Perceptual Functions), and b2102 (Quality of Vision)—the I-CVI was 0.83, which still exceeded the acceptable threshold of 0.78. This finding indicates that the scale comprehensively covers the construct it is intended to measure. The S-CVI for the ICCSDS was 0.978, which far exceeded the minimum standard of 0.90, indicating good content validity.

#### 3.5.2 Construct validity

The construct validity of the ICCSDS was analyzed via EFA. For the assessments conducted by the rehabilitation physician, the Kaiser–Meyer–Olkin (KMO) measure was 0.887, and Bartlett’s test of sphericity yielded an approximate chi-square value of 1621.280 (*p* < 0.01), with a total cumulative variance 75.69% being explained. For the assessments conducted by the swallowing therapist, the KMO value was 0.848, and Bartlett’s test yielded an approximate chi-square value of 1179.518 (*p* < 0.01), with a total cumulative variance of 73.65% being explained. These findings provided preliminary evidence supporting the construct validity of the ICCSDS; however, the factor structure should be interpreted cautiously given the relatively small sample size (Table 3).

#### 3.5.3 Criterion-related validity

In this study, the GUSS was used as a practical criterion measure for evaluating criterion-related validity. The total ICCSDS scores assessed by the rehabilitation physician and the swallowing therapist were 36.92 ± 16.30 and 32.41 ± 14.56, respectively. The Pearson correlation coefficients between the ICCSDS and GUSS scores were 0.790 and 0.683, respectively (both *p* < 0.01), indicating moderate-to-strong criterion-related validity of the ICCSDS against the GUSS (Table 3).

## 4 Discussion

### 4.1 ICCSDS item analysis

In this study, the SDs of all the ICCSDS items were substantially greater than 0.5, indicating that patient scores were not clustered around a specific value but instead showed considerable variability. This variability suggests that the items of the ICCSDS are sensitive to capturing differences among patients in the corresponding functional or structural domains. For example, the rehabilitation physician reported the greatest variability for b1302 (Appetite), with a SD of 1.84, whereas the swallowing therapist reported the greatest variability for e110 (Products or substances for personal consumption), with a SD of 1.71. In addition, b117 (Intellectual functions) showed a relatively large SD (1.62). These differences reflect important clinical phenomena. Items b1302 and b117 represent broader cognition-related categories, whereas e110 represents an environmental factor category. Their assessments may, to some extent, depend on raters’ subjective judgments regarding patients’ cognitive status and their interpretation of food characteristics. In clinical practice, rehabilitation physicians tend to interpret cognitive impairment from a pathophysiological perspective, whereas swallowing therapists are more focused on how cognitive function affects feeding behavior, participation in rehabilitation training, and the impact of different food consistencies on swallowing performance. Such differences in professional perspectives may lead to variations in the interpretive anchors applied by the two raters when assessing these items.

The scores of the environmental factor categories (e110, e340, e410, and e580) were negative, which is consistent with the ICF coding convention in which positive qualifiers indicate barriers and negative qualifiers indicate facilitators. These findings also suggest that under similar inpatient rehabilitation conditions, the two raters demonstrated a high degree of consistency in determining whether environmental factors functioned as facilitators or barriers. However, because only two raters were included in the present study, the generalizability of these differences remains limited. Future studies should recruit a larger number of raters with diverse professional backgrounds to further validate cross-disciplinary scoring consistency.

Most items (e.g., b117 and e110) had similar SDs between the two raters. This similarity may be because the scoring anchors for these items have been relatively standardized in clinical practice or because these items are based on relatively objective and observable conditions with limited room for subjective interpretation. Consequently, raters from different professional backgrounds are more likely to reach consistent judgments. In contrast, several items (e.g., b1302 and s320) showed more pronounced differences in score variability between the raters. This variability may be attributed to differences in clinical perspectives: rehabilitation physicians may emphasize the pathophysiological basis of functional impairment, whereas swallowing therapists may focus more on the impact of functional deficits on actual feeding behaviors and daily participation. Therefore, the two groups of professionals may assign different degrees of relative importance to the functional impairments reflected by specific items. Furthermore, structural impairment assessments (e.g., s320, Structure of the mouth) do not always correspond linearly with functional performance, which may further contribute to discrepancies in scoring.

Furthermore, on the basis of the critical ratio method, significant differences were observed between the high-score and low-score groups for all the ICCSDS items. These findings indicate that each item of the ICCSDS has good discriminative ability and can effectively distinguish patients with different levels of dysphagia severity, supporting the retention of all the items in the scale.

### 4.2 ICCSDS demonstrates good reliability

Reliability refers to the stability, consistency, and dependability of results obtained from the same scale by different evaluators. Cronbach’s α coefficient is typically used to evaluate the internal consistency of a scale. In this study, the Cronbach’s α coefficients for the ICCSDS, as assessed by the rehabilitation physician and swallowing therapist, were 0.864 and 0.829, respectively. Both values exceed 0.8, indicating excellent internal consistency, homogeneity, and stability and confirming that the scale is a reliable tool for assessing swallowing dysfunction in stroke patients. The ICCSDS employs unified operational items from quantitative standards, analyzes category definitions, and extracts concise and clear key points for inquiry. This process aims to reduce uncertainty among raters and patients regarding the understanding of ICF categories and qualifiers, as well as inconsistencies in language comprehension and expression, thereby facilitating operation and understanding and improving interrater reliability (23, 24). High interrater reliability is a crucial prerequisite for the clinical application and promotion of the ICCSDS.

In the present study, the weighted kappa coefficients for the vast majority of the ICCSDS items exceeded 0.80, indicating a high degree of agreement between the two raters with different professional backgrounds in their interpretation of the items. These findings were consistent with the overall ICC results and further supported the reliability of the scale for interdisciplinary clinical use. Notably, items such as d550 (Eating), d560 (Drinking), and d630 (Preparing meals) had weighted kappa coefficients greater than 0.90. These greater values may be attributable to the fact that these categories involve overt and readily observable behaviors, with relatively clear assessment criteria and greater consistency in interpretation among raters. However, a small number of items, including b510 (Ingestion functions), b5101 (Biting), b5102 (Chewing), and e580 (Health services, systems, and policies), showed relatively low weighted kappa coefficients (<0.50). For b5101 (Biting) and b5102 (Chewing), which may be related to the complexity of the motor patterns involved and the lack of objective quantitative measures, such as bite force or masticatory electromyography. Assessments of these functions rely largely on clinical observation and subjective judgment, which may result in inconsistent thresholds for classifying mild, moderate, and severe impairments among raters. In particular, b510 (Ingestion functions) is a composite item encompassing multiple components of the eating process, and different raters may place different emphases on these components. In contrast, the closely related oral item b51050 (Oral swallowing) had a very high weighted kappa coefficient, which may be attributable to the relatively clear scale and the ease with which this aspect can be understood and assessed by patients and raters.

The relatively low agreement for e580 (Health services, systems, and policies) may reflect its inherently subjective nature. In practice, raters may primarily infer this item from the patient’s type of health insurance, while differences in rater familiarity with specific healthcare policies may contribute to greater variability in assessment. For e410 (Individual attitudes of immediate family members), the relevant information is largely derived from family members’ participation and cooperation, as well as from the manner in which the raters conduct interviews, making the assessment more susceptible to subjective bias. The item b117 (Intellectual functions) represents a composite cognitive domain. Patients with dysphagia may also present with dysarthria and mild cognitive impairment, which could affect the accuracy of verbal expression and consequently influence the assessment of this item. Differences in professional training and clinical priorities may further lead to variation in the interpretive anchors applied by raters when evaluating these items.

Overall, the ICCSDS demonstrated good interrater reliability, with approximately two-thirds of the items showing high levels of agreement, supporting the overall reliability of the scale. With respect to the six items with moderate agreement, future studies could develop more specific anchor definitions and a standardized scoring manual to reduce interprofessional discrepancies in scoring and further improve the applicability of the scale across diverse clinical settings.

### 4.3 ICCSDS demonstrates good validity

The validity of a scale primarily reflects the degree of agreement between the assessment results and the intended outcomes (25). The evaluation indicators used in this study included content validity, construct validity, and criterion-related validity. Six experts with extensive clinical experience in swallowing function assessment were invited to rate content relevance. The I-CVI was 0.83 for three items—b1303 (Craving), b156 (Perceptual Functions), and b2102 (Quality of Vision)—and 1 for all the other items. The S-CVI was 0.978. Items b1303 (Craving), b156 (Perceptual Functions), and b2102 (Quality of Vision) are associated with differences regarding food in the preswallowing phase and have less impact on the pharyngeal phase. Therefore, some experts hold divergent views on the importance of these items, mainly because they have less influence on the pharyngeal phase. Overall, however, the content validity of the items in this assessment scale is good.

Numerous studies have shown that the use of GUSS scores not only aids in formulating early swallowing function training plans but also allows patients, families, and healthcare staff to intuitively understand patients’ swallowing status across foods of different consistencies (26, 27). In this study, the GUSS score was used as the criterion to validate the correlation between the ICCSDS measurement indicators and the GUSS score. The criterion-related validity coefficients for assessments by the rehabilitation physician and swallowing therapist were 0.790 and 0.683, respectively. The correlation coefficient for both was approximately 0.70, providing preliminary support for the good criterion-related validity of the ICCSDS. However, the GUSS is a bedside screening tool rather than an instrumental diagnostic reference standard such as the VFSS or FEES. Therefore, the observed correlations provide supportive but incomplete evidence of criterion-related validity and should not be interpreted as validation against a gold standard. In future studies, ICCSDS scores should be compared with objective swallowing measures obtained using VFSS or FEES.

Furthermore, in this study, EFA was performed to evaluate the construct validity of the 21 ICCSDS categories. The results showed that the KMO values obtained by both assessors were greater than 0.80, and Bartlett’s test of sphericity was statistically significant. The cumulative variance explained exceeded 70% for both assessments, providing preliminary support for the construct validity of the scale. However, considering the relatively small sample size and the single-center design, the factor structure of the ICCSDS requires further validation in larger and more diverse samples.

With respect to item refinement, on the basis of the 21 core categories extracted previously, we further revised the descriptions of the abstract and potentially ambiguous categories through discussion with experienced clinical experts. These revisions aimed to transform the original items into simpler and more intuitive descriptions, thereby facilitating accurate interpretation by clinical users. In addition, the original ICF qualifiers (0–4), which have relatively unclear boundaries for defining the severity of functional impairment, were converted into a numerical scoring system more familiar to clinicians. Clear anchor definitions for each severity level were established to improve the practicality and validity of the ICCSDS in clinical applications. These modifications were intended to enhance the operational feasibility of the scale and improve scoring consistency, aligning with the requirements for evaluating the reliability and validity of ICF generic core sets in clinical practice (28). Nevertheless, whether these modifications provide additional benefits for improving reliability and validity requires further confirmation in future studies.

The ICF is a classification system for functioning, disability, and health, providing standardized language for functional assessment and serving as a foundational framework for health-related policy, including prevention, treatment, and rehabilitation. The ICF reflects not only disease severity but also the patient’s activity and participation capacity within this state of dysfunction. It reflects a person-centered, holistic functional state and is a critical tool for clinical functional assessment (24, 29).

In recent years, the ICF framework has been increasingly emphasized in the assessment and management of dysphagia. In 2022, M Renom-Guiteras, in a study optimizing the holistic semiqualitative assessment of oropharyngeal dysphagia to improve rehabilitation treatment, emphasized the need to follow the ICF’s biopsychosocial framework model (30). In 2023, Klara Movander’s team combined the Dysphagia Outcome and Severity Scale with ICF concepts, validating the effectiveness of the ICF framework in professional collaborative assessment (31). Jennifer Moloney’s team extensively studied patient-reported outcomes for poststroke dysphagia, mapping relevant scales to the ICF structure, which not only enhanced the validity of the ICF core elements scale to assess dysphagia but also provided a scientific basis for its clinical application (32). In 2025, Zhang Xia’s team assessed swallowing function in stroke patients using the ICF framework and proposed that ICF assessment tools can help clinical teams predict rehabilitation progress, rationally plan treatment pathways, and reduce healthcare costs (33). Wu Yacen’s team used the ICF scale and item response theory models to construct the iSWAL-Performance scale specifically for dysphagia, which contains 50 ICF items. This scale not only demonstrated good reliability and validity but also intuitively reflected the alignment between a patient’s swallowing ability and assessment items, facilitating adjustments to rehabilitation strategies on the basis of the patient’s specific functional level (34).

### 4.4 Limitations and future directions

This study has several limitations. The small, single-center sample may limit the stability and generalizability of the exploratory factor structure; thus, larger multicenter samples and confirmatory factor analysis are needed. GUSS served as a pragmatic clinical comparator rather than an instrumental gold standard, and validation against the VFSS or FEES is warranted. Interrater reliability was assessed using only one rehabilitation physician and one swallowing therapist, and the four-hour interval between different raters did not permit classical test–retest assessment. Future studies should include multiple raters and repeated assessments by the same rater. TOR-BSST-based recruitment and exclusion of patients with severe communication/cognitive impairment, acute stroke, or tracheostomy or those receiving outpatient care may also limit generalizability. The cross-sectional design further precluded the assessment of responsiveness, minimal detectable changes, and minimally clinically important differences. Future longitudinal studies should investigate the responsiveness of the ICCSDS to rehabilitation-related improvement or deterioration and determine its minimal detectable change and minimal clinically important difference. Finally, the relevance of several less direct swallowing-related ICF categories requires further investigation. Overall, these findings provide preliminary psychometric evidence supporting the potential utility of the ICCSDS, but larger, multicenter, longitudinal validation is needed.

## 5 Conclusion

The ICCSDS demonstrated favorable psychometric properties, including internal consistency, content validity, construct validity, and criterion-related validity, providing preliminary support for its clinical applicability in patients with dysphagia.

## Data Availability

The raw data supporting the conclusions of this article will be made available by the authors, without undue reservation.
