# Supplementary material for: Reliability and validity of the ICF core elements scale for swallowing function assessment in stroke patients: a single-center study from Hunan, China
Source: Front Neurol. 2026 Oct 7;17:1863539. doi: 10.3389/fneur.2026.1863539 (PMC13688877; doi:10.3389/fneur.2026.1863539)
Supplement: Supplementary file 1 [file Table_1.DOCX]

**ICF Core Category Scale for Dysphagia in Stroke**

| Category, code and item of ICF | Question | Score | | | | | | | | | | |  |
| --- | --- | --- | --- | --- | --- | --- | --- | --- | --- | --- | --- | --- | --- |
|  |  | No impairment | | Mild impairment | | Moderate impairment | | Severe impairment | | | Complete impairment | |  |
| **Body functions** |  |  | |  | |  | |  | | |  | |  |
| b117 (Intellectual functions) | Do you recognize the food you are eating and know how it should be eaten? | 0 | | 1 | | 2 | | 3 | | | 4 | |  |
| b1301(Motivation) | Do you have the motivation to eat? | 0 | | 1 | | 2 | | 3 | | | 4 | |  |
| b1302(Appetite) | Is your appetite the same as usual? | 0 | | 1 | | 2 | | 3 | | | 4 | |  |
| b1303 (Craving) | Do you have a strong preference for a particular food, such that you lose your appetite when it is unavailable? | 0 | | 1 | | 2 | | 3 | | | 4 | |  |
| b140 (Attention functions) | Can you eat without being distracted by your surroundings? | 0 | | 1 | | 2 | | 3 | | | 4 | |  |
| b156 (Perceptual functions) | Do the color, aroma, or taste of food affect your appetite? | 0 | | 1 | | 2 | | 3 | | | 4 | |  |
| b1644(Insight) | Do you know how to address your swallowing difficulties? | 0 | | 1 | | 2 | | 3 | | | 4 | |  |
| b2102 (Quality of vision) | Have you noticed any changes in your vision? Do these changes affect your eating? | 0 | | 1 | | 2 | | 3 | | | 4 | |  |
| b250(Taste functions) | Do you feel that your sense of taste is impaired? | 0 | | 1 | | 2 | | 3 | | | 4 | |  |
| b510 (Ingestion functions) | Do you experience difficulty swallowing? | 0 | | 1 | | 2 | | 3 | | | 4 | |  |
| b5101(Biting) | Do you have difficulty biting or tearing large pieces of food? | 0 | | 1 | | 2 | | 3 | | | 4 | |  |
| b5102(Chewing) | Do you have difficulty opening your mouth or chewing food? | 0 | | 1 | | 2 | | 3 | | | 4 | |  |
| b51050 (Oral swallowing) | Can you swallow food smoothly by mouth? | 0 | | 1 | | 2 | | 3 | | | 4 | |  |
| **Body structures** |  |  | |  | |  | |  | | |  | |  |
| S320 [Structure of the mouth (teeth, gums)] | Do you have any oral health problems or diseases? | 0 | | 1 | | 2 | | 3 | | | 4 | |  |
| **Activities and participation** |  |  | |  | |  | |  | | |  | |  |
| d550 (Eating) | **P:** Is your ability to eat affected when using assistive feeding devices and/or with assistance from a caregiver? | 0 | | 1 | | 2 | | 3 | | | 4 | |  |
|  | **C:** Is your ability to eat affected when eating without assistive feeding devices and/or caregiver assistance? | 0 | | 1 | | 2 | | 3 | | | 4 | |  |
| d560 (Drinking) | **P:** Is your ability to drink liquids affected when using assistive devices and/or with assistance from a caregiver? | 0 | | 1 | | 2 | | 3 | | | 4 | |  |
|  | **C:** Is your ability to drink liquids affected when drinking without assistive devices and/or caregiver assistance? | 0 | | 1 | | 2 | | 3 | | | 4 | |  |
| d630 (Preparing meals) | **P:** Can you complete grocery shopping and meal preparation with assistance? | 0 | | 1 | | 2 | | 3 | | | 4 | |  |
|  | **C:** Can you complete grocery shopping and meal preparation independently? | 0 | | 1 | | 2 | | 3 | | | 4 | |  |
|  |  | Complete facilitator | Substantial facilitator | | Moderate facilitator | Mild facilitator | Neither facilitator nor barrier | | Mild barrier | Moderate barrier | Severe barrier | Complete barrier | |
| **Environmental factors** |  |  |  | |  |  |  | |  |  |  |  | |
| e110 (Products or substances for personal consumption) | To what extent do changes in food (e.g., different textures or amounts of sauce) facilitate or hinder your eating? | -4 | -3 | | -2 | -1 | 0 | | 1 | 2 | 3 | 4 | |
| e340 (Personal care providers and personal assistants) | To what extent does the support and attitude of your caregiver facilitate or hinder your eating? | -4 | -3 | | -2 | -1 | 0 | | 1 | 2 | 3 | 4 | |
| e410 (Individual attitudes of immediate family members) | To what extent does the support and attitude of your family facilitate or hinder your eating? | -4 | -3 | | -2 | -1 | 0 | | 1 | 2 | 3 | 4 | |
| e580 (Health services, systems, and policies) | To what extent do your health insurance policies facilitate or hinder your access to swallowing rehabilitation therapy? | -4 | -3 | | -2 | -1 | 0 | | 1 | 2 | 3 | 4 | |

**Total score:** ______

**Note:** The **C (capacity)** scores for the Activities and participation items are not included in the total score.
